# Cancer: Genetic Basis of UVB Sensitivity

**Published:** 2006-05

**Authors:** Victoria McGovern

More than 1 million new U.S. cases of basal cell carcinoma (BCC) and squamous cell carcinoma (SCC) will be diagnosed this year, according to the American Cancer Society, and most will be highly curable. New melanoma will be diagnosed in only about 62,000 Americans, but will be far more fatal if not caught early; five-year survival for melanoma that has aggressively spread is only 16%. A study in the 21 December 2005 *Journal of the National Cancer Institute* now shows a genetic difference between melanoma patients and those with other skin cancers: melanoma patients’ chromosomal DNA (chromatin) suffers less damage than other skin cancer patients’ when cells are irradiated with ultraviolet B (UVB) light, the part of UV that causes sunburn.

The work, led by epidemiologist Qingyi Wei of the University of Texas M.D. Anderson Cancer Center, examined how susceptibility to large-scale DNA damage in the form of chromosome breaks differed among patients with different types of skin cancer. “At the chromosomal level, BCC and SCC patients seem more sensitive in terms of the number of chromosomal breaks per cell,” Wei says. In earlier work, his laboratory established that people with melanoma and BCC are less efficient at repairing UV-induced DNA damage than are cancer-free controls; he’s now working on a similar study on SCC.

BCC and SCC have clear dose–response curves with sun exposure, says Nick Hayward, a human geneticist at the Queensland Institute of Medical Research in Brisbane. In contrast, melanoma is more associated with acute intermittent doses. “Instead of going out and getting sunlight every day,” he says, “people who get melanoma tend to be those who go to the beach without a tan, stay out too long, and get absolutely cooked.”

Although most skin cancers derive from either melanocytes or keratinocytes, the assay looks for physical breaks in the chromosomes of lymphocytes—nucleated blood cells—taken from skin cancer patients and cancer-free controls to estimate an individual’s sensitivity to UVB. Blood cells are collected, grown in culture, irradiated under controlled conditions, and allowed to recover for a day for cellular repair to occur. Then researchers count gaps in the cells’ chromatin. Cancer patients whose cells showed more chromosome breaks after UVB irradiation were 3 times more likely than the general population to have BCC or SCC, but were not more likely to have melanoma.

“One thing that’s satisfying about this study is that it fits nicely with some of the known genetic and environmental causes, particularly of BCC, but also of SCC,” says Graham Mann, a geneticist at the University of Sydney’s Westmead Institute for Cancer Research. “It’s been known for years that people with a severe familial form of BCC are very prone to BCC formation after ionizing radiation, presumably because they get much more chromosomal damage.”

The assay is not on its way to development as a diagnostic, but rather adds to our understanding of the genetics of cancer. “If you want to diagnose patients,” Wei says, “you have to have a thorough, specific assay. You don’t want to make mistakes.”

And in case sunbathers think they are safe against melanoma, they should remember that UVA radiation can still damage the DNA in melanocytes.

